# Supplementary material for: Association between the COVID-19 pandemic and mental health in very old people in Sweden
Source: PLoS One. 2024 Apr 2;19(4):e0299098. doi: 10.1371/journal.pone.0299098 (PMC10986980; doi:10.1371/journal.pone.0299098)
Supplement: Attachment — Submitted filename: Response to Reviewers2.docx [file pone.0299098.s002.docx]

The second response to Reviewers

We thank once again the reviewers for their time and effort in reviewing our manuscript! Please find our response below.

**Reviewer #1**

1.**Topic:** *The authors could be more specific in the topic by stating the nation name, as the study is primarily focused on the context of Sweden*.

RESPONSE: We are not sure what “topic” refers to, but we have changed the title and aim so that it now states “in Sweden”. We hope this settles the concern.

2. **Result:** *In order to analyze the statistical findings, the commentary of the tables in the result section needs to be expanded, especially section 3.4 - social factors.*

RESPONSE: Thank you for pointing our attention to this aspect. We have made changes to section 3.4 in the results and also in parts of the discussion where we added a paragraph, particularly for discussing these findings.

**Reviewer #2:**

Reviewer #2 ask us to improve the answer to five previous concerns and two new ones, and we are happy to try to make a better answer!

1. **Previous point 8** *Data and Methodology: 250 people filled out the survey (line 103), but the numbers in Table 1, Table 2, and Table 3 do not match up in the rows and columns where they should. If the row data is 247, how is it split between columns 75 and 175? And it is also applicable for other numbers in row.*

*Data and methodology: cleaning of data and study sample*

RESPONSE: We are still not sure if we have understood your concern correctly, but we have now added information on the denominator in Tables 1, 2, and 3.

For instance: Residential status (residing alone), among those with negative impact on mental health by COVID-19 there were 38 residing alone and 75 were able to answer, and among those without negative impact 79 residing alone of 169. That means that of (38+79=) 117 were residing alone and (75+169=) 244 who were able to answer the question. We now have presented all the values and the reader can make Chi-square calculations if desired to check our numbers.

2. **Previous points 9, 10, and 12** *Does the author have any studies that show how to combine subjective responses with five groups into two? Do author has any reference to club the category Not enough to explain why the categories of responses might be merged.*

RESPONSE: The use of dichotomization has been discussed over many years (1-3). It can be used to simplify data analyses and interpretation and to be able to compare. There are drawbacks such as loss of information, the cut-offs might not be obvious, different cut-offs can lead to different conclusions, decreased precision, etc. We have added to the manuscript a reason for using dichotomization in the method section and in the limitations discussed drawback of such procedure.

There are manuscripts that for instance combine five answer alternatives to two, evaluation of one’s health (4, look in Table 1).

1. Ben-Shakhar G. A further study of the dichotomization theory in detection of information. Psychophysiology. 1977 Jul;14(4):408-3. PMID: 882621.
2. Altman DG, Royston P. The cost of dichotomising continuous variables. BMJ. 2006 May 6;332(7549):1080. PMID: 16675816;
3. Fedorov V, Mannino F, Zhang R. Consequences of dichotomization. Pharm Stat. 2009 Jan-Mar;8(1):50-61. PMID: 18389492.
4. Tak E, Staats P, Van Hespen A, Hopman-Rock M. The effects of an exercise program for older adults with osteoarthritis of the hip. J Rheumatol. 2005 Jun;32(6):1106-13. PMID: 15940775.
3. **Previous point 15** *OR, LL, and UL estimates or highly questionable. [(sample mean) - (constant) x (SEM)] to [(sample mean) + (constant) x (SEM)] could be used to determine the Confidence Interval. Therefore, it may be closer to the mean value of CI.*

RESPONSE: This was a difficult concern for the authors, so we asked for help from an experienced statistician who gave this answer to provide in this response to Reviewer #2:

”As for the comment on how confidence intervals for odds ratios should be calculated, we agree that the suggested expression is correct for coefficients in a linear model. However, since we use odds ratios as is customary in logistic regression, this expression is not correct.”

We therefore conclude that the output generated from SPSS statistical software is accurate.

4. **New point 1** *I-ADL and P-ADL: The author does not provide specific details for these categories.*

RESPONSE: We thank the reviewer for this comment and agree that the reader could benefit from knowing the background for I-ADL and P-ADL. The following two sentences were included:

“To be deemed as independent in I-ADL the participants had to be independent in all the following activities: cleaning, shopping, transport, and cooking. To be deemed independent in P-ADL, the participants had to be independent in all the following activities: bathing, dressing, toileting, transfer, continence, and eating.”

5. **New point 2** *Even under table 4, section 3.5 Predictors of deteriorating mental health is not adequately discussed in detail.*

RESPONSE: Thank you for pointing our attention to this aspect. We have made changes to section 3.4 in the results and also in parts of the discussion where we added a paragraph, particularly for discussing these findings.
